# Effect of Exercise Prescription Implementation Rate on Cardiovascular Events

**DOI:** 10.3389/fcvm.2021.753672

**Published:** 2022-02-03

**Authors:** Li-Yue Zhu, Min-Yan Li, Kun-Hui Li, Xiao Yang, Yi-Yong Yang, Xiao-Xia Zhao, Ting Yan, Meng-Meng Li, Si-Qi Luo, Mu-Lan Zhang, Jin-Zi Su

**Affiliations:** ^1^Rehabilitation Center, Zhejiang Hospital, Hangzhou, China; ^2^Department of Rehabilitation Medicine, The School of Health, Fujian Medical University, Fuzhou, China; ^3^Department of Cardiology, The First Affiliated Hospital of Fujian Medical University, Fuzhou, China; ^4^Fuyang District Traditional Chinese Medicine Hospital, Hangzhou, China

**Keywords:** cardiac rehabilitation, cardiovascular diseases, exercise prescription, cardiovascular events, coronary heart disease

## Abstract

**Background:**

Exercise prescription of cardiac rehabilitation (CR) is vital in patients with cardiovascular diseases (CVDs) and those carrying high risk for CVDs. However, the relation between the implementation rate of exercise prescription and cardiovascular events (CVEs) is unclear.

**Design and Methods:**

In this retrospective study, using the administration data from the Rehabilitation Center in a hospital, patients aged ≥18 years with CVDs were consecutively enrolled from November 2018 to May 2021. Patients were divided into the high execution group (HEG) and low execution group (LEG) depending on whether they completed more than half the time of the exercise prescriptions. Baseline characteristics, ultrasonic cardiogram, cardiopulmonary exercise test, follow-up data, and CVEs were collected.

**Results:**

The mean age of the 197 CR patients was 61.8 ± 13.7 years and the mean follow-up duration was 10.9 ± 4.2 months. Among them, 15 patients suffered CVEs: 4 in the HEG and 11 in the LEG. The incidence of CVEs showed significant differences between HEG and LEG (chi-square test). Free-event survival analysis using Kaplan–Meier survival plots showed that patients in LEG had poor survival. Cox proportional hazards regression analysis revealed that the prescription implementation rate was an independent predictor of CVEs.

**Conclusions:**

Our study suggested a significant effect of exercise prescription execution rate on the occurrence of CVEs. Further, the HEG of exercise prescription was associated with lower CVDs.

## Introduction

Cardiovascular diseases (CVDs) are a serious global health concern. A systematic analysis for the Global Burden of Disease Study 2017 reported that CVDs affect approximately 485.6 million people worldwide (1). According to the National Center for Cardiovascular Diseases' most recent estimates, there are 330 million cases of CVDs in China, accounting for 40% of all deaths (2). With the development and progression of new treatment strategies, cardiac rehabilitation (CR) has received a lot of attention. The American Heart Association (AHA), the American College of Cardiology (ACC), and the European Society of Cardiology (ESC) have all recognized and advocated CR as a vital aspect of modern cardiology (3, 4). At present, CR is still in its early stages of growth in China. Chinese cardiologists have proposed the Five Prescriptions rule, which integrate pharmacological prescription, exercise prescription, diet prescription, psychological prescription, and smoking-cessation prescription to provide comprehensive and holistic management and care for patients with CVDs.

Exercise training is regularly identified as the cornerstone of comprehensive CR in international guidelines (5). Exercise has been classified as Type 1A evidence in various CR guidelines and consensus in the fight against CVDs risk. The importance of exercise in CR is self-evident (5–7). Exercise training has been found in clinical research to improve blood pressure and cholesterol control in persons at risk of CVDs (8, 9). Patients with coronary artery disease (CAD) and heart failure may also see considerable improvements (10–12). Furthermore, exercise improves clinical prognosis by lowering illness incidence, overall mortality, cardiovascular disease-related mortality, rehospitalization rates, and the need for revascularization (13, 14).

A patient's prognosis is heavily influenced by the extent of exercise prescription that is carried out. However, in our clinical work, we frequently discover that even when physicians and therapists target individualized exercise prescriptions for patients, the exercise prescription implementation (i.e., type, frequency, volume, intensity of exercise, duration of sessions and programs, goals, and progressive training adaptations) varies significantly from patient to patient (15, 16). Given the link between exercise prescription rates and cardiovascular events (CVEs), a better knowledge of their relationship could help doctors improvise treatment strategies, improve patients' outcomes, and reduce medical costs. Thus, we investigated the prognostic effects of exercise prescription implementation on CVEs in CR patients to suggest directions for future work.

## Materials and Methods

### Study Participants

A total of 197 patients with CR from November 8, 2018 to May 11, 2021 in the Rehabilitation Center of Zhejiang Hospital, Zhejiang Province, were included in this study, which was approved by the Ethics Committee of the hospital (No. 2014-KA-8). The inclusion criteria were (1) age≥18 years; (2) myocardial infarction, CAD, post percutaneous coronary intervention, hypertension, diabetes mellitus, heart failure, dilated cardiomyopathy, and/or other high-risk CVDs; and (3) the need for CR. The exclusion criteria were as follows: (1) symptomatic systolic blood pressure >200 mmHg or diastolic blood pressure >110 mmHg at rest or a drop in blood pressure >20 mmHg after sitting-up; (2) severe aortic stenosis; (3) acute systemic disease or fever; (4) uncontrolled severe atrial or ventricular arrhythmias, uncontrolled significant sinus tachycardia (>120 beats/min); (5) uncontrolled heart failure or third-degree atrioventricular block without a pacemaker; (6) active pericarditis or myocarditis, thrombophlebitis, recent thromboembolism, and ST-segment depression or elevation (>2 mm) at rest; (7) severe exercise system abnormalities that can limit exercise capacity and other metabolic abnormalities such as acute thyroiditis, hypokalemia, hyperkalemia, or hypovolemia; (8) pregnancy; and (9) severe mental illness or dementia in patients making them unable to cooperate. After enrollment, medical history including basic personal information (age, sex, current medical history, past history, and education) was collected, and an informed consent form was signed was by all participating patients.

### Ultrasonic Cardiogram

Cardiac function was assessed using an ultrasonic cardiogram with Color Doppler Ultrasonography (Vivid E9, GE, USA) at a scanning frequency of 3.5 MHz and parasternal left ventricular long-axis views to measure left atrial internal diameter, left ventricular internal diameter, septal thickness, and posterior left ventricular wall thickness, as well as left atrioventricular valve flow spectrograms. Furthermore, early diastolic E-peak flow, E-peak deceleration time, late diastolic A-peak flow velocity, LV early/late diastolic peak flow velocity ratio (E/A), and LV isovolumic diastolic time were all evaluated at end-expiration velocity.

### Cardiopulmonary Exercise Test

CPET (Cosmed, Rome, Italy) and OMNIA cardiorespiratory diagnostic software were used to assess aerobic capacity. CPET exercise program selected sub-maximum quantity exercise or symptom-limited exercise test by using a cardiopulmonary exercise tester (17). Lung function was measured before exercise, and oxygen consumption and other indicators, 12-lead electrocardiogram (ECG), and blood pressure were continuously monitored during exercise. After a 3-min resting period, the bicycle was pedaled at 55–65 r/min for 3 min without load, and then the power was increased in 10–20 W/min increments until symptom limitation was reached or the target exercise volume was reached. After achieving maximum workout power, a 5–10 min recuperation time was implemented. The heart rate, oxygen uptake, oxygen pulse, blood pressure, metabolic equivalent, and power of the patient were all continuously recorded. The cardiorespiratory analysis system analyzes sample data and generates reports that include information on the patient's resting and anaerobic threshold conditions.

### Exercise Prescription Execution Ability and Grouping

Based on the CPET results, aerobic exercise training generates exercise prescriptions and intensities. During the inpatient phase, the CR physician formulated individual exercise prescriptions for the patients in the CR group, and the therapists instructed the implementation of rehabilitation exercises according to the exercise prescriptions: (1) Warm-up exercises: The muscles, joints, and cardiovascular system were warmed up for 10–15 min with low-intensity warm-up exercises. (2) Workouts were conducted in the hospital ward or exercise therapy room. Exercise methods included walking, power bicycles, dumbbells, joint stretching, 8 or 24 forms of Taijiquan, or exercise gymnastics. Real-time cardiac monitoring was performed with a BeneView T5 patient monitor or POLAR heart rate meter. Heart rate, rhythm, blood pressure, and fatigue level were closely observed and recorded. (3) Relaxation period: 5–10 min following the last recovery exercise. (4) Precautions: blood pressure was monitored before and after exercise, and ECG was monitored throughout. Patients were asked to exercise at an intensity corresponding to an RPE of 11–14 (18). The duration of each exercise was 30–45 min, once a day. The rehabilitation center was equipped with the above-mentioned Sports Art C52r power bicycle, Sports Art 6300HR running table, and other CR exercise equipment, with soothing background music, as well as oxygen, monitors, defibrillators, resuscitation drugs, and other resuscitation equipment. Concurrently, group health education was conducted twice a week, combined with individual education. The content format was mainly slide lectures combined with reading pamphlets to help correct risk factors for coronary heart disease, including guidance on smoking cessation, dietary guidance, psychological assessment, and emotional management to help establish a healthy lifestyle.

**Figure 1:**
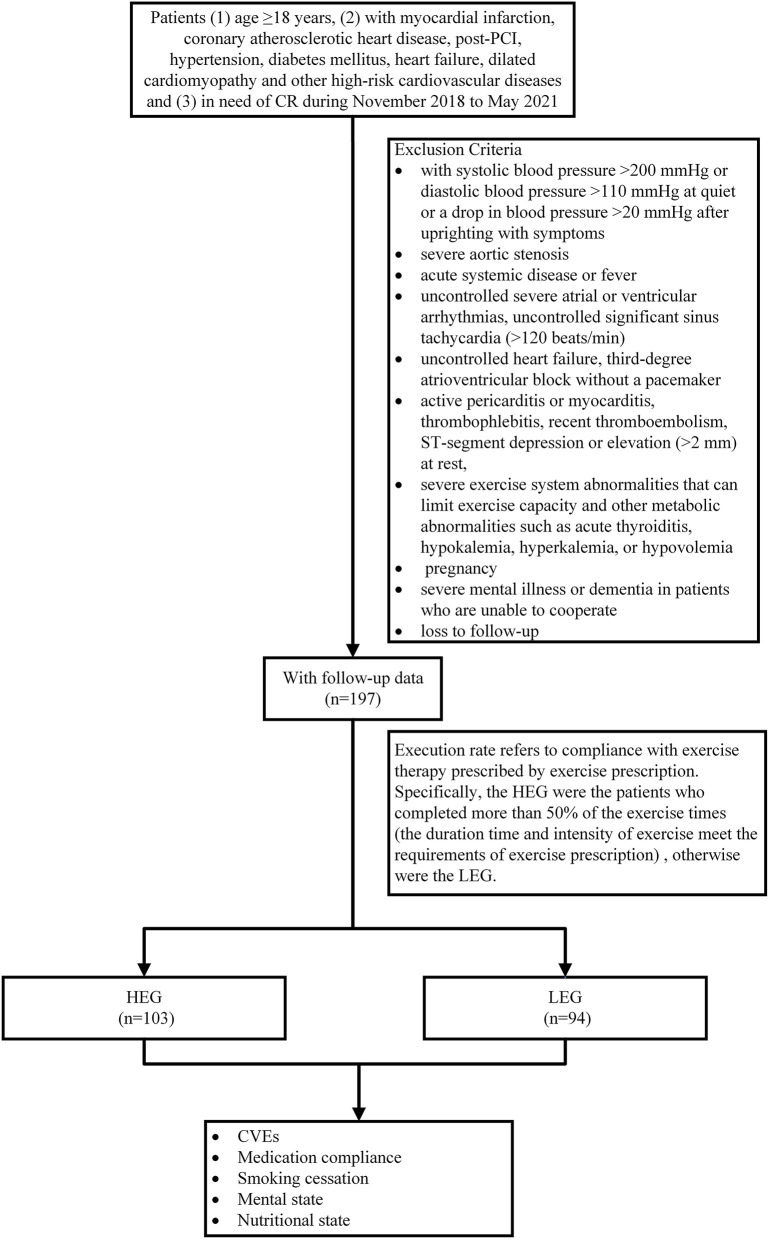
Study design flowchart. PCI, percutaneous coronary interventions; CR, cardiac rehabilitation; HEG, high execution group; LEG, low execution group; CVEs, cardiovascular diseases.

During the discharge phase, outpatient CR was conducted. Outpatient rehabilitation was performed three times a week, 36 times a cycle according to exercise prescriptions, with an assessment once every 4 weeks and adjustment of exercise prescriptions. Home CR was performed after 3 months. Some low-risk patients were discharged from the hospital for home CR, with telephonic follow-up every 2 weeks. Specifically, the high execution group (HEG) (*n* = 104) comprised patients who completed >50% of the exercise times (the duration time and intensity of exercise met the requirements of exercise prescription); all other patients were classified under the low execution group (LEG) (*n* = 93) (Figure 1).

### Definition of CHD, PCI, CVEs, Medication Compliance, Smoking Cessation, Mental State, and Nutritional State

CHD: The Cardiovascular Disease Branch of the Chinese Medical Association's “Guide to the rational use of drugs in the primary level for stable coronary artery disease” was consulted for the definition of diagnostic criteria for coronary artery disease (19).

PCI: The “Chinese guidelines for percutaneous coronary interventions (2016)” were referred to for all percutaneous coronary procedures (20).

CVEs: These were defined as the composite endpoint of acute myocardial infarction recurrence, acute angina recurrence, acute heart failure, atrial fibrillation, stroke and death.

Medication compliance: The extent to which an individual consistently adhered to the medication recommended by the healthcare provider.

Smoking cessation: The smokers giving up their dependence on nicotine. Successful cessation was when the patient quit and did not resume smoking during the observation period.

Mental state: The complete characteristics of psychological activities in a certain period of time, including anxiety, depression, tension, relaxation, sadness, and joy.

Nutritional state: It is closely related to food intake, digestion, absorption, and metabolism, and is used to reflect the level of material energy provided by all activities of the organism, and its status can be used as one of the criteria to identify health and disease, which is divided into good nutrition, malnutrition, and overnutrition.

### Study Outcome and Follow-Up

All participants had access to standardized medicine as well as health promotion and risk-factor management. The primary outcome was recurrent CVEs status, and secondary outcomes included status of medication compliance, smoking cessation, mental state, and nutritional status. Information for the inpatient phase was derived from the case system, while information for the home rehabilitation phase was obtained by professional staff during telephonic follow-up visits or outpatient follow-up visits. Medication adherence was assessed using the Morisky 8-item Medication Adherence Questionnaire (MMAS-8). Psychological status was assessed using the Patient Health Questionnaire-9 (PHQ-9) and the Generalized Anxiety Disorder (GAD-7). Smoking status was assessed using the Fagerstrm Test for Nicotine Dependence (FTND). Nutritional status was assessed using the Inpatient Nutrition Risk Screening Scale 2002 (NRS2002). To avoid temporal bias, all individuals were followed-up until death or July 7, 2021.

### Statistical Analyses

SPSS 26.0 statistical software (IBM Corporation, Armonk, NY, USA) was used for analysis. Continuous variables were expressed as M (Q1, Q3) using the Mann–Whitney U rank sum test, while categorical variables were expressed as percentages (%) using the chi-square test. Event-free survival was analyzed using Kaplan–Meier hypothesis testing between patients with HEG or LEG. Curves were compared with the log-rank test and the Breslow test. The association between exercise prescription implementation rate and CVEs was evaluated by Cox proportional hazards model. Univariate Cox analysis was first performed to screen all prognostic factors. Variables with clinical significance or *P* < 0.05 in the univariate analysis were selected for multivariate Cox analysis (backward method). Differences were considered statistically significant at *P* < 0.05.

## Results

### Clinical Characteristics of Patients

The mean age of the 197 CR patients was 61.8 ± 13.7 years, and the mean follow-up duration was 10.9 ± 4.2 months. The proportion of HEG and LEG were 52.3 and 47.7% respectively. In demographic data, biochemical indicators, concomitant disorders, and ECG, there were no significant differences between the two groups. CPET was also not statistically significant between the two groups (Table 1).

**Table 1:** Baseline characteristics of patients.

**Characteristic**	**HEG (*n =* 103)**	**LEG (*n* = 94)**	***p-*value**
Age, years	60.0 (52.0–69.0)	64.5 (54.0–73.3)	0.234
Male	78 (75.7)	72 (76.6)	0.887
BMI, kg/m^2^	25.1 (23.2–27.2)	24.2 (22.4–26.7)	0.156
Smoke	23 (22.3)	26 (27.7)	0.387
Follow-up time, months	12.0 (7.0–14.0)	11.0 (7.0–14.0)	0.107
SBP, mmHg	130.0 (117.0–139.0)	126.0 (114.8–143.8)	0.644
DBP, mmHg	77.0 (69.0–87.0)	75.0 (66.0–84.5)	0.091
**Laboratory data**			
TC, mmol/L	4.2 (3.4–5.1)	3.9 (3.2–4.9)	0.307
TG, mmol/L	1.4 (1.0–1.9)	1.4 (1.0–1.8)	0.342
HDL, mmol/L	1.1 (0.9–1.2)	1.0 (0.8–1.2)	0.052
LDL, mmol/L	2.3 (1.6–2.8)	2.3 (1.7–2.9)	0.421
**Comorbidities and interventions**			
CHD	62 (60.2)	48 (51.1)	0.197
PCI	50 (80.6)	36 (75.0)	0.477
Anti-platelet agents	56 (90.3)	39 (81.3)	0.169
Statins	56 (90.3)	43 (89.6)	0.898
Hypertension	74 (71.8)	57 (60.6)	0.096
ACEI or ARB	51 (68.9)	37 (64.9)	0.628
β-blocker	41 (55.4)	28 (49.1)	0.475
CCB	27 (36.5)	19 (33.3)	0.708
Diuretic	15 (20.3)	14 (24.6)	0.558
Diabetes	34 (33.0)	22 (23.4)	0.135
Anti-diabetic	26 (76.5)	18 (81.8)	0.634
**Ultrasonic cardiogram**			
Aod, mm	31.3 (29.0–33.0)	30.1 (28.0–33.0)	0.124
LAD, mm	35.0 (33.0–38.0)	35.9 (33.0–39.1)	0.361
LVDd, mm	50.2 (47.3–52.2)	50.9 (47.4–52.9)	0.305
LVDs, mm	32.0 (29.0–33.9)	33.0 (28.8–34.0)	0.278
IVS, mm	10.1 (8.8–11.0)	10.0 (8.8–10.9)	0.763
LVPW, mm	9.6 (8.7–10.2)	10.1 (8.6–11.2)	0.070
FS, %	35.7 (33.0–38.7)	35.4 (32.8–39.3)	0.989
EF, %	63.9 (60.0–69.0)	64.9 (61.1–70.0)	0.454
E1, cm/s	68.2 (59.0–75.0)	70.0 (56.0–81.3)	0.215
A1, cm/s	83.3 (77.0–92.0)	82.0 (71.0–90.0)	0.565
E/A	0.8 (0.7–0.9)	0.8 (0.7–1.0)	0.379
**Cardiopulmonary function exercise test**			
Exercise duration, s	380.0 (306.0–455.0)	383.0 (303.8–456.3)	0.935
Peak work load, w	88.0 (60.0–110.0)	84.0 (65.3–114.8)	0.895
Peak VO_2_, mL/min	1348.0 (1029.0–1643.0)	1319.5 (1068.0–1710.0)	0.880
Peak VO_2_/kg, mL/ (min·kg)	19.2 (16.3–22.4)	19.6 (16.4–23.6)	0.436
Peak O_2_/HR, mL/beat	10.8 (8.8–13.1)	11.1 (9.2–12.9)	0.670
Peak mets	5.5 (4.6–6.4)	5.6 (4.6–6.6)	0.547
AT, mL/min	1032.0 (824.0–1277.0)	947.0 (828.5–1150.8)	0.192

*Continuous variables are expressed as median (IQR) and analyzed by the Mann–Whitney U rank sum test. Categorical variables are expressed as number and percentage and analyzed by the chi-square test. HEG, high execution group; LEG, low execution group; BMI, body mass index; SBP, systolic blood pressure; DBP, diastolic blood pressure; TC, total cholesterol; TG, triglyceride; HDL-C, high-density lipoprotein; LDL-C, low-density lipoprotein; CHD, coronary atherosclerotic heart disease; PCI, percutaneous coronary intervention; ACEI, angiotensin-converting enzyme inhibitor; ARB, angiotensin receptor antagonist; CCB, calcium channel blocker; Aod, aortic diameter; LAD, left atrial diameter; LVDd, left ventricular end diastolic diameter; LVDs, left ventricular systolic diameter; IVS, interventricular septal thickness; LVPW, left posterior wall; FS, fraction shortening; EF, left ventricular ejection fraction; E/A, ratio of peak E to peak A; Peak VO_2_, peak oxygen uptake; Peak VO_2_/kg, peak oxygen uptake per kilogram of body weight; Peak O_2_/HR, oxygen pulse; Peak Mets, peak metabolic equivalent; AT, anaerobic threshold*.

### Follow-Up of Patients

A total of 197 patients were followed-up. Regular medication and mental health were substantially better in the follow-up situation than in the LEG (regular medication: 99.0 vs. 87.2%, *P* = 0.001 mental health: 97.1 vs. 81.9%, *P* < 0.001) (Table 2).

**Table 2:** Outcome of follow-up.

**Characteristic**	**HEG (*n* = 103)**	**LEG (*n* = 94)**	***p-*value**
**Outcome of follow-up**			
Regular medication	102 (99.0)	82 (87.2)	0.001
Smoking cessation	13/23 (56.5)	16/26 (61.5)	0.721
Mental health	100 (97.1)	77 (81.9)	<0.001
Weight stability	86 (83.5)	69 (73.4)	0.084

*Categorical data were expressed as number and percentage and analyzed by the chi-square test. HEG, high execution group; LEG, low execution group*.

### Event-Free Survival

**Figure 2:**
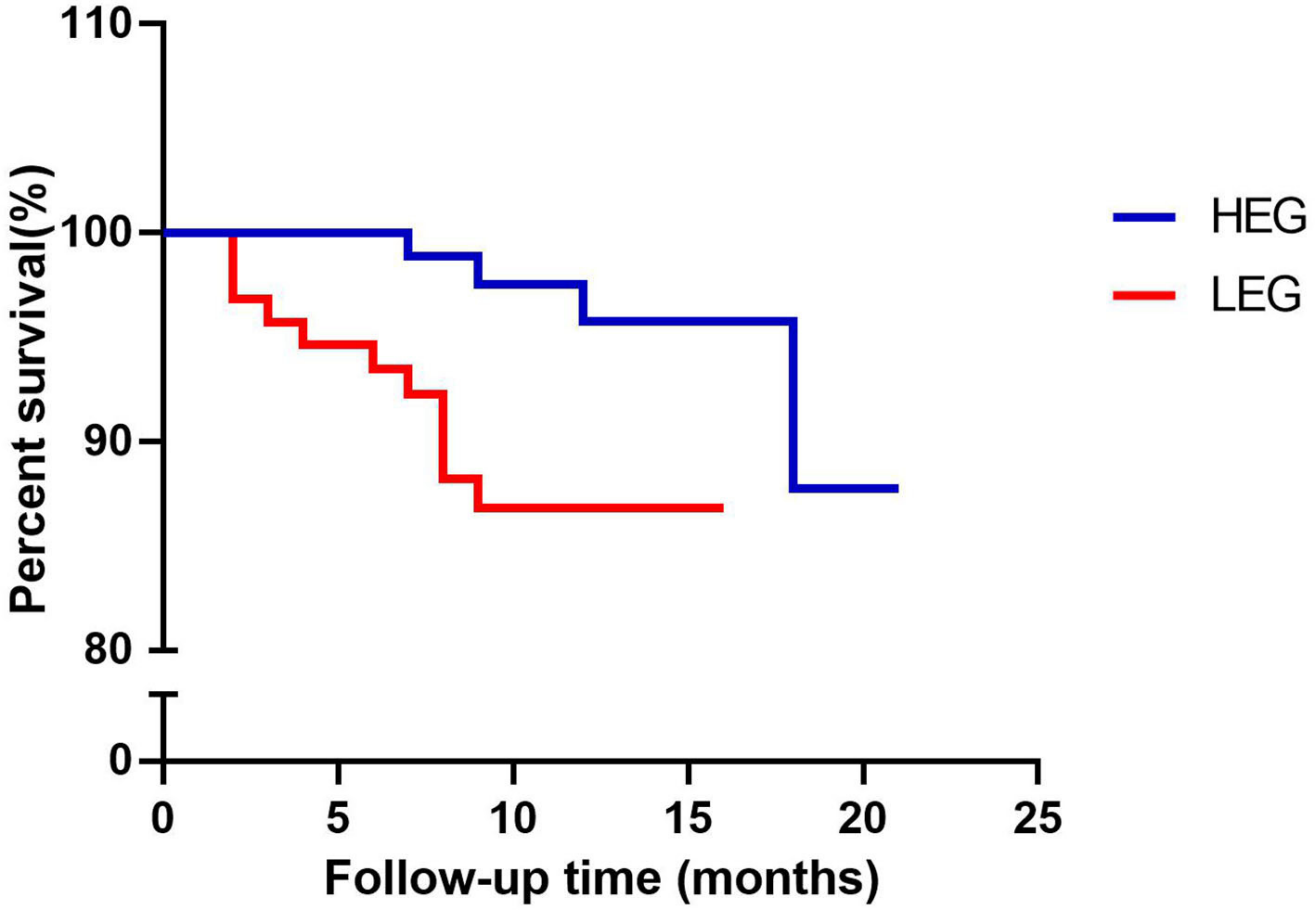
Kaplan–Meier curves of event-free survival with follow-up time. HEG, high execution group; LEG, low execution group.

CVEs were shown to be considerably more common in LEG than in HEG (*P* = 0.039) (Table 3). During follow-up, four HEG patients (3.9%) suffered at least one cardiovascular event, including one acute myocardial infarction, two acute anginas, and one stroke. During follow-up, eleven LEG patients (11.7%) had at least one cardiovascular event, including one acute myocardial infarction, four acute anginas, two acute heart failures, one atrial fibrillation, and three deaths (Table 3). The Kaplan–Meier hypothesis testing method was used to analyze event-free survival. The Breslow test revealed a significant difference (*P* = 0.008) between the two groups, and the log-rank test also revealed a significant difference (*P* = 0.016) (Figure 2).

**Table 3:** Incidence of cardiovascular events in study patients.

**Characteristic**	**HEG (*n* = 103)**	**LEG (*n* = 94)**	***p*-value**
CVEs	4 (3.9)	11 (11.7)	0.039
Acute myocardial infarction	1	1	
Acute angina	2	4	
Acute heart failure		2	
Atrial fibrillation		1	
Stroke	1		
Death		3	

*Categorical data were expressed as number and percentage and analyzed by the chi-square test. HEG, high execution group; LEG, low execution group; CVEs, cardiovascular events*.

### Cox Proportional Hazards Regression Analysis for CVEs

In univariate Cox analysis, age, smoking, interventricular septal thickness (IVS), and low execution of exercise were significant factors (*P* < 0.05) (Table 4). Considering their clinical importance, comorbidities and unregular medication were also selected for multivariate Cox analysis (backward method). Model 1 was adjusted for age, smoking, IVS, and low execution of exercise. Model 2 was adjusted for the variables in model 1 plus CHD, hypertension, and diabetes. Model 3 was adjusted for the variables in model 1 plus unregular medication. In all three Cox models, execution of exercise was found to be an independent predictor of CVEs (Table 5).

**Table 4:** Univariate Cox analysis of proportional risks for CVEs.

**Variable**	**β**	**Standard error**	**HR (95% CI)**	**Wald**	***p-*value**
Age	0.039	0.019	1.040 (1.002–1.080)	4.165	**0.041**
Male	−0.803	0.760	0.448 (0.101–1.987)	1.116	0.291
BMI	−0.111	0.084	0.895 (0.759–1.055)	1.752	0.186
Smoke	1.052	0.518	2.865 (1.037–7.914)	4.121	**0.042**
SBP	0.015	0.015	1.015 (0.986–1.044)	1.003	0.317
DBP	0.000	0.020	1.000 (0.962–1.040)	0.000	0.996
TC	−0.334	0.249	0.716 (0.440–1.166)	1.798	0.180
TG	−0.311	0.349	0.733 (0.370–1.451)	0.795	0.372
HDL	−0.420	1.082	0.657 (0.079–5.437)	0.151	0.698
LDL	0.093	0.298	1.097 (0.612–1.966)	0.098	0.755
CHD	0.449	0.550	1.567 (0.533–4.604)	0.666	0.414
Hypertension	−0.890	0.519	0.411 (0.149–1.135)	2.946	0.086
Diabetes	−0.830	0.761	0.436 (0.098–1.936)	1.191	0.275
Aod	0.081	0.056	1.085 (0.973–1.210)	2.136	0.144
LAD	0.040	0.035	1.041 (0.972–1.115)	1.336	0.248
LVDd	0.051	0.052	1.052 (0.950–1.165)	0.956	0.328
LVDs	0.009	0.044	1.009 (0.925–1.101)	0.044	0.833
IVS	−0.279	0.104	0.756 (0.617–0.927)	7.211	**0.007**
LVPW	−0.153	0.132	0.858 (0.662–1.112)	1.338	0.247
FS (%)	−0.003	0.043	0.997 (0.915–1.085)	0.006	0.937
EF (%)	0.001	0.030	1.001 (0.944–1.061)	0.001	0.975
E1	−0.002	0.014	0.998 (0.971–1.026)	0.019	0.890
A1	−0.016	0.014	0.984 (0.957–1.012)	1.223	0.269
E/A	0.321	0.648	1.378 (0.387–4.911)	0.245	0.621
Exercise duration	0.001	0.002	1.001 (0.996–1.005)	0.074	0.786
Peak work load	−0.006	0.007	0.994 (0.981–1.008)	0.720	0.396
Peak VO_2_	0.000	0.001	1.000 (0.999–1.001)	0.003	0.953
Peak VO_2_/kg	−0.022	0.047	0.979 (0.892–1.074)	0.209	0.648
Peak VO_2_/HR	0.063	0.095	1.065 (0.885–1.281)	0.439	0.508
Peak MET	−0.047	0.183	0.954 (0.666–1.366)	0.067	0.796
VO_2_ @AT	0.000	0.001	1.000 (0.998–1.001)	0.109	0.742
Unregular medication	0.105	1.038	1.111 (0.145–8.501)	0.010	0.919
Low execution of exercise	1.438	0.652	4.213 (1.175–15.107)	4.872	**0.027**
Non-smoking cessation	0.174	0.646	1.119 (0.336–4.218)	0.073	0.788
Mental unhealth	0.917	0.652	2.501 (0.697–8.971)	1.978	0.160
Weight instability	1.014	0.531	2.755 (0.974–7.796)	3.647	0.056

*CVEs, cardiovascular events; SBP, systolic blood pressure; DBP, diastolic blood pressure; TC, total cholesterol; TG, triglyceride; HDL-C, high density lipoprotein; LDL-C, low density lipoprotein; CHD, coronary atherosclerotic heart disease; PCI, percutaneous coronary intervention; ACEI, angiotensin-converting enzyme inhibitor; ARB, angiotensin receptor antagonist; CCB, calcium channel blocker; Aod, aortic diameter; LAD, left atrial diameter; LVDd, left ventricular end diastolic diameter; LVDs, left ventricular systolic diameter; IVS, interventricular septal thickness; LVPW, left posterior wall; FS, fraction shortening; EF, left ventricular ejection fraction; E/A, ratio of peak E to peak A; Peak VO_2_, peak oxygen uptake; Peak VO_2_/kg, peak oxygen uptake per kilogram of body weight; Peak O_2_/HR, oxygen pulse; Peak Mets, peak metabolic equivalent; AT, anaerobic threshold. Bold values indicates p <0.05*.

**Table 5:** Multivariate Cox analysis of proportional risks for CVEs.

**Model**	**Variable**	**β**	**HR (95%CI)**	***p*-value**
1	Low execution of exercise	1.387	4.003 (1.062–15.095)	**0.041**
	Age	0.052	1.054 (1.012–1.097)	**0.011**
	Smoke	1.365	3.914 (1.280–11.967)	**0.017**
	IVS	−0.324	0.723 (0.584–0.895)	**0.003**
2	Low execution of exercise	1.446	4.247 (1.067–16.911)	**0.040**
	Age	0.049	1.050 (1.006–1.097)	**0.026**
	Smoke	1.340	3.820 (1.252–11.657)	**0.019**
	IVS	−0.301	0.740 (0.594–0.921)	**0.007**
	CHD	0.560	1.751 (0.581–5.275)	0.320
	Hypertension	−0.659	0.517 (0.181–1.481)	0.219
	Diabetes	−0.450	0.637 (0.134–3.028)	0.571
3	Low execution of exercise	1.363	3.906 (1.024–14.906)	**0.046**
	Age	0.052	1.054 (1.012–1.097)	**0.011**
	Smoke	1.395	4.036 (1.292–12.606)	**0.016**
	IVS	−0.329	0.720 (0.581–0.893)	**0.003**
	Unregular medication	0.335	1.398 (0.169–11.575)	0.756

*CVEs, cardiovascular events; IVS, interventricular septal thickness; CHD, coronary atherosclerotic heart disease. Bold values indicates p <0.05*.

## Discussion

In this study, we followed-up 197 CR patients. We developed individualized exercise prescriptions for each patient based on their unique characteristics, and then classified them into HEG and LEG based on the exercise prescription's completion. We discovered through the study's analysis that varied exercise prescriptions had a substantially positive impact on the occurrence of CVEs. Moreover, prescription implementation rate was found to be an independent predictor of CVEs. The impact of better exercise prescription implementation on the incidence of CVEs has to be researched further, according to the study findings.

In our research, we discovered that patients who followed their exercise prescriptions at a higher rate had fewer CVE episodes. The results can be explained as two aspects. First, we looked at the baseline data and follow-up results and discovered that while there were no noticeable variations in the baseline, the follow-up outcomes differed substantially. When compared to the LEG, the HEG exhibited superior adherence in terms of regular medication-taking and mental health maintenance. Medication compliance largely reflects a patient's attitude toward medical advice. Moreover, good-users of drugs may also have better compliance with medical treatments and healthy lifestyle choices than users with poor compliance (21). Negative mood, whether overt or covert, is extremely likely to be one of the main non-cardiac reasons for lack of motivation and exercise discontinuance in CR (22, 23). CR is a self-contained and active process. Patients who adhere to their treatment plans are more likely to engage in physical activity. We paid more attention to the occurrence of CVEs. There were 4 CVEs in the HEG and 11 in the LEG among the endpoint events. The larger case numbers, more CVEs variants, and more severe occurrences that occurred in the LEG can be summarized. On the part of the event-free survival during the following-up time, the results of log-rank test reflected the long-term outcome, while the Breslow test showed the short-term outcome. When we combined the baseline data with the follow-up outcomes, we can see that the HEG with the same condition had considerably better adherence and a significantly lower risk and severity of CVEs than the LEG.

In a retrospective study from Belgium, which divided patients with CAD into supervised and unsupervised groups, the supervised group performed reasonably standardized multidisciplinary exercise rehabilitation for 3 months longer than the unsupervised group. The results showed that in patients with CHD, the supervised group had considerably fewer hospitalizations for adverse CVEs (24). Longer supervised exercise training, stricter adherence to pharmaceutical prescriptions, more healthy food counseling, and greater psychological distraction were all hypothesized to be connected with the clinical advantages found in this study. The findings of this study were similar to those of ours, which may have profited from their multidisciplinary collaboration. This study's findings were similar to ours in terms of trend; however, our statistical findings were not as significant as theirs, which could be attributed to their bigger sample size and multidisciplinary joint intervention (25).

High-risk, older cardiac patients were placed into two groups in a single-blind randomized clinical trial on CR in the Netherlands: normal treatment and CR intervention. There were no significant differences in readmission and mortality rates between the two groups after 6 months, and the event rate was slightly lower in the usual care group than in the CR intervention group, despite the CR intervention group receiving more standardized and systematic comprehensive rehabilitation (26). This study did not come up with the same results as ours. This could be because the high-risk elderly have a higher chance of mortality and CVEs; hence, there was no meaningful difference between the two groups. As a result, the impact of exercise prescription execution must be investigated further.

Our study has some limitations. First, because this was a retrospective case study rather than a randomized controlled trial, the possibility of selection bias impacting the results of the study cannot be ruled out. Second, the study had a single-center design with a small sample size, which could limit the generalizability of the results. Third, the follow-up information was insufficient. In the future, we will more rigorously standardize patient enrollment criteria, develop randomized controlled clinical trials for specific disease populations, and conduct longitudinal and prospective analyses to further clarify the effect of different exercise prescription execution rates on CVEs.

To summarize, although we found a substantial effect of exercise prescription execution rate on unfavorable CVEs, this study has limitations and the potential advantages of high CR exercise prescription execution rates must be verified in more refined, bigger, and longer-term trials. Furthermore, to adopt better management techniques and enhance clinical prognosis, efforts should be made to discover significant elements that can promote patient compliance.

## Data Availability Statement

The original contributions presented in the study are included in the article/supplementary material, further inquiries can be directed to the corresponding authors.

## Ethics Statement

Written informed consent was obtained from the individual(s) for the publication of any potentially identifiable images or data included in this article.

## Author Contributions

L-YZ, J-ZS, and M-LZ conceived the idea, designed the study, and established the methodology. XY, X-XZ, and TY were responsible for follow-up, data collection, and collation. M-YL and K-HL wrote and revised the manuscript. M-ML and S-QL were in charge of software, literature retrieval, and visualization. M-YL, K-HL, and Y-YY carried out the analyses. All authors reviewed and approved the manuscript prior to submission.

## Funding

This work was supported by the Science and Technology Project of Fujian Province, China (No. 2020Y0023), Medical Science and Technology Project of ZheJiang Province (No. 2014KA8), and Zhejiang Province Public Welfare Technology Application Research Project (No. 2015C33121).

## Conflict of Interest

The authors declare that the research was conducted in the absence of any commercial or financial relationships that could be construed as a potential conflict of interest.

## Publisher's Note

All claims expressed in this article are solely those of the authors and do not necessarily represent those of their affiliated organizations, or those of the publisher, the editors and the reviewers. Any product that may be evaluated in this article, or claim that may be made by its manufacturer, is not guaranteed or endorsed by the publisher.
